# Which Factors Are Associated with Persistence of Depressive and Anxiety Symptoms in Patients Affected by Atopic Dermatitis despite 2-Year Treatment with Dupilumab?

**DOI:** 10.3390/jcm13071980

**Published:** 2024-03-29

**Authors:** Silvia Mariel Ferrucci, Simona Tavecchio, Alessandro Ceresa, Luisa Angileri, Emilio Berti, Angelo Valerio Marzano, Massimiliano Buoli

**Affiliations:** 1 Dermatology Section, Fondazione IRCCS Ca’ Granda, Ospedale Maggiore Policlinico, 20122 Milan, Italy; silvia.ferrucci@policlinico.mi.it (S.M.F.);; 2 Department of Pathophysiology and Transplantation, University of Milan, 20122 Milan, Italy; massimiliano.buoli@unimi.it; 3 Department of Neurosciences and Mental Health, Fondazione IRCCS Ca’ Granda, Ospedale Maggiore Policlinico, Via F. Sforza 35, 20122 Milan, Italy

**Keywords:** Atopic Dermatitis, dupilumab, anxiety, depression, treatment resistance

## Abstract

**Background**: Atopic Dermatitis (AD) is a prevalent inflammatory skin disease whose course is often complicated by the presence of concomitant anxiety and depressive disorders. Dupilumab has been demonstrated to be largely effective in AD. The aims of the present study were to (1) to verify the effectiveness of 2-year dupilumab treatment on the depressive and anxiety symptoms of patients affected by AD and (2) to identify predictors of the persistence of psychiatric symptoms despite maintenance treatment with dupilumab. **Methods**: A total of 331 patients with severe AD were assessed at baseline and at different times over 2 years by a large set of rating scales, including the Eczema Area and Severity Index (EASI), the Hospital Anxiety and Depression Scale (HADS), and the Dermatology Life Quality Index (DLQI). Paired sample *t*-tests were performed to verify the effectiveness of dupilumab on the severity of AD and mental health items. Two binary logistic regression models were then used to identify the predictors of the persistence of clinically significant depression and anxiety, defined by a score ≥ 8 on each sub-scale of the HADS. **Results**: After 2 years of treatment with dupilumab, the patients benefited, showing a significant improvement in both the dermatological disease and comorbid depression/anxiety (*p* < 0.001 for all scales). Overall, 17.5% and 13% of patients, respectively, reported residual depressive and anxiety symptoms after the 2-year treatment with dupilumab. The baseline predictors of the persistence of clinically significant depressive symptoms after the 2-year treatment with dupilumab were found to be a higher body mass index (BMI) (*p* = 0.012), a lower impact of dermatological disease on quality of life (*p* = 0.015), and more severe depressive symptoms (*p* < 0.01), while for anxiety, the only predictor was found to be female gender (*p* = 0.03). **Conclusions**: Using a multidisciplinary approach, at baseline, dermatologists should more closely monitor patients who are at a greater risk of maintaining residual psychiatric symptoms despite therapy, such as those with more severe depressive symptoms and those who are overweight.

## 1. Introduction

Atopic Dermatitis (AD) is a prevalent and disabling condition which often occurs in comorbidity with other medical conditions [1]. Psychiatric disorders are frequently manifested by patients suffering from AD, complicating the course and the management of dermatological illness [2]. Several psychiatric conditions are reported by subjects affected by AD, including attention deficit/hyperactivity disorder, autism spectrum disorder [3], and eating disorders in children/adolescents [4] and mood disorders, schizophrenia [5], and alcohol [6] and internet addiction in adults [7]. Affective disorders (anxiety and depression) represent one of the most frequent psychiatric comorbidities, as reported by different authors [8,9], including in a previous study by our group [10]. Different factors explain the vulnerability of patients with AD to mood/anxiety disorders, including the biological alterations shared by the dermatological and psychiatric illness [11], such as over-inflammation [12,13], metabolic abnormalities [2,14], or the accumulation of advanced glycation end products in the skin [15,16]. Psychosocial factors such as discomfort and discrimination related to skin lesions can also contribute to the deterioration of patients’ mental health [17]. Of note, the presence of psychiatric disorders complicates the course of AD in terms of illness severity [18], suicidal risk [19], and treatment compliance [20].

Dupilumab is a human monoclonal antibody that blocks interleukin 4 and 13 signaling, therefore acting on the main disease mechanism of AD [21]. Dupilumab is efficacious in the treatment of moderate–severe AD [22,23] and has been demonstrated to be more well tolerated than conventional immunosuppressive drugs (e.g., cyclosporine) [21]. As over-inflammation characterizes both depression and a number of autoimmune diseases, including AD, in recent years, different research groups have wondered whether anti-inflammatory drugs could be effective for the management of mood disorders, especially in the case of other concomitant medical conditions involving immunity [24,25]. A recent manuscript reported that dupilumab is more effective than a placebo in improving the sleep disorders of patients affected by AD [26]. These findings are in line with the results of a publication by our group that showed a decrease in anxiety and depressive disorders in patients who received at least one year of dupilumab treatment than those who were candidates for or recently started therapy with this compound [7].

In light of these considerations, the objectives of this study were (1) to verify the amelioration of depressive and anxiety symptoms, as well as of dermatological disease, with dupilumab treatment and (2) to identify the predictors of persistence of depressive/anxiety disorders after long-term treatment with dupilumab (2 years).

## 2. Materials and Methods

Patients with moderate–severe AD followed up at the Dermatology Outpatient Clinic, Fondazione IRCCS Ca’ Granda Ospedale Maggiore Policlinico, Milan, Italy, were selected for the purposes of the present study, which was approved by the local Ethics Committees. The inclusion criteria were (1) subjects who had a diagnosis of AD and (2) patients who completed a 2-year dupilumab treatment. The exclusion criteria were (1) pregnancy or (2) an inability to give consent to participate into the study. The presence of medical comorbidities or a psychiatric diagnosis did not represent reasons for exclusion from the study, with the exception of conditions that could hamper treatment tolerability, like severe conjunctivitis. Written informed consent was obtained from each participant of this study.

Data about the following demographic and clinical variables at baseline were collected: age, age at onset, body mass index (BMI), gender, and AD type (intrinsic/extrinsic).

The following rating scales, used to assess illness severity and the presence of affective symptoms, were administered at baseline and at different times until 24 months (at 1, 4, 8, 12, 16, and 20 months): the Hospital Anxiety and Depression Scale (HADS), the Patient-Oriented Eczema Measure (POEM), the Dermatology Life Quality Index (DLQI), the Sleep Quality Numeric Rating Scale (SQ-NRS), the Itch Numerical Rating Scale (itch-INRS), Physician Global Assessment (PGA), the Eczema Area and Severity Index (EASI), and the Atopic Dermatitis Control Tool (ADCT). Total plasma immunoglobulin E (IgE) levels were measured at the same time points. The properties of these tools (with the exception of ADCT) were described in other manuscripts published by our research group [7,10]. The HADS is a scale widely used to evaluate affective symptoms in patients with medical comorbidities and consists of two subscales (one for depression and one for anxiety) [27]. The presence of clinically significant depressive or anxiety symptoms was defined as a HADS score for each sub-scale ≥8 [27]. The SQ-NRS is a simple single-item tool designed to assess one’s quality of sleep the previous night [28]. The POEM, itch-INRS, PGA, and EASI are all instruments used in clinical practice to assess the severity of AD [10]. The DLQI was the first scale to be introduced to evaluate quality of life in patients affected by skin diseases [29]. Finally, ADCT is a self-rated tool that consists of six questions (each with a range score 0–4) to evaluate the different aspects of AD: a score ≥7 points was identified as the optimum threshold to identify patients affected by AD without clinical remission [30].

**Figure 1:**
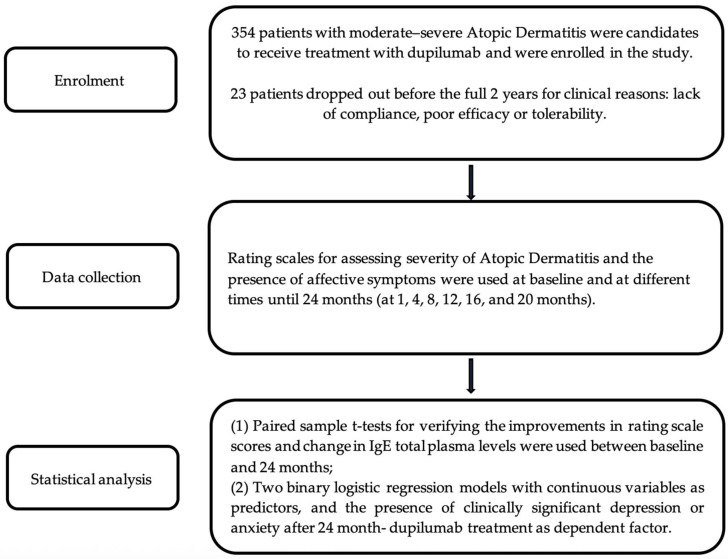
Flow diagram of the study.

Descriptive statistic analyses were performed (mean and standard deviation for continuous variables and frequencies for qualitative ones). Paired sample *t*-tests were used to verify the improvements in rating scale scores and change in IgE total plasma levels between baseline and 24 months after starting the treatment with dupilumab. Two binary logistic regression models were used to perform logistic regression, with continuous variables as predictors and the presence of clinically significant depression or anxiety after 24 months of dupilumab treatment as the dependent factor. The goodness of fit of the models was assessed by Hosmer–Lemeshow tests. The level of statistical significance was set at *p* ≤ 0.05 (Figure 1).

## 3. Results

Three hundred and fifty-four patients were initially assigned to the dupilumab treatment group, but 23 (6.5%) dropped out before the full 2 years for clinical reasons (lack of compliance, poor efficacy, or tolerability). The sample included in this analysis therefore consisted of 331 patients with an age at the beginning of dupilumab treatment of 37.03 years (±16.96), an age at onset of 11.12 years (±18.97), a BMI of 24.46 (±10.50), and a duration of illness of 26.03 years (±15.70): 183 (55.3%) were males, 300 (90.6%) had an extrinsic type AD, 57 (17.2%), and 42 (12.7%) presented depressive and anxiety symptoms after 2 years of dupilumab treatment. The participants received their baseline visit between 2018 and 2021. During the study, current SARS-CoV-2 infection was ascertained in 20 patients by molecular swab; four subjects had suffered from a past infection, as documented by the presence of antibodies. One hundred and forty-eight participants received at least one vaccine dose.

Data about the baseline clinical variables of the total sample are reported in Table 1.

Only five patients were followed up by outpatient psychiatric clinics at the beginning of dupilumab treatment: two had a diagnosis of Generalized Anxiety Disorder, one of Major Depressive Disorder, and two of Panic Disorder. Only one participant was receiving treatment with citalopram, and another was taking part in an ongoing psychotherapy regime. The rating scale scores and serum IgE levels of the total sample at baseline and after 24 months of dupilumab treatment are reported in Table 2. The following adverse events related to treatment with this compound were developed by these patients: eosinophilia (N = 43, 13%), conjunctivitis (N = 71, 21.4%), headache (N = 1, 0.3%), oral herpes (N = 3, 0.9%), and molluscum contagiosum (N = 2, 0.6%). These adverse events were mild/moderate, and the discontinuation of the treatment was not necessary.

The patients showed a significant amelioration in all rating scale scores (related to both AD and anxiety/depressive disorders) after 24 months of dupilumab treatment (all *p* < 0.001) (Table 2).

With regard to the persistence of depressive symptoms after 24 months of dupilumab treatment, the model was reliable (Hosmer and Lemeshow Test: χ^2^ = 7.203, *p* = 0.515), allowing for a correct classification of 81.3% of cases. The presence of depressive symptoms was predicted by a higher baseline BMI (odds ratio (OR): 1.157, 95% confidence interval (CI): 1.033–1.296, *p* = 0.012), a lesser impact of dermatological disease on quality of life—DLQI—scores (OR: 0.898, 95% CI: 0.823–0.979, *p* = 0.015), and more severe depressive symptoms—HADS-D—scores (OR: 1.253, 95% CI: 1.086–1.446, *p* = 0.002) (Table 3).

With regard to the persistence of anxiety symptoms after 24 months of dupilumab treatment, the model was reliable (Hosmer and Lemeshow Test: χ^2^ = 5.872, *p* = 0.662), allowing for a correct classification of 85.6% of cases. The presence of anxiety symptoms was predicted only by female gender (OR: 2.466, 95% CI: 1.257–4.837, *p* = 0.034) (Table 4).

## 4. Discussion

As expected, long-term treatment with dupilumab (24 months) resulted in significant amelioration in all clinical aspects of AD, confirming the findings of the available literature on this topic [31]. Of note, this compound had a significant beneficial effect on sleep disorders and affective symptoms, thus supporting recently published data [7,26,32]. Furthermore, a recent study reported that treatment with dupilumab improves depressive symptoms in other atopic conditions such as asthma [33]. The effectiveness of this compound on mood and anxiety disorders could be secondary to the remission of skin lesions, with an improvement in self-esteem [34]. Alternatively, the anti-inflammatory properties of the compound could have a direct impact on psychiatric disorders as well as on the course of AD [35]. Furthermore, the restoration of regular circadian rhythms, disrupted by nocturnal itch, could promote recovery from depressive and anxiety disorders [36]. The study of the effectiveness of immunity modulators on psychiatric disorders is still at a pioneering stage and calls for more in-depth future research [37]. In addition, it has not been totally clarified which role is attributable to the anti-inflammatory properties of available antidepressants in explaining their effectiveness [38].

As previously mentioned, the remission of depressive and anxiety symptoms in patients with AD is crucial, as the response to antibody treatment may be reduced in the presence of these types of symptoms, as shown in samples of patients affected by asthma [39]. Of note, a minority but significant part of patients with AD manifest affective disorders after long-term treatment with dupilumab. This finding could suggest that other factors beyond inflammation may contribute to make subjects affected by AD vulnerable to affective disorders such as cortisol release dysregulation, altered lipid metabolism, and a sedentary lifestyle associated with overweight [40]. Of note, with regard to the identified predictors of persistence of depression in our sample, several studies indicate that a higher BMI is associated with more severe mood disorders [41,42] as a result of common pathophysiological processes underlying obesity and depressive illness (e.g., leptin/insulin dysregulation or intestinal microbiome alterations) [43]. A similar association was reported for BMI and AD, formulating the hypothesis that hypertrophied adipocytes contribute to a pro-inflammatory state [44]. Anti-inflammatory drugs such as dupilumab ameliorate immune system regulation and the skin microbiome, but they probably have fewer effects on the above-mentioned factors that link depressive disorders and obesity [45]. This consideration is supported by the observation in our study of the persistence of depressive symptoms in patients whose dermatological disease has a lesser impact on their quality of life; this aspect can seem paradoxical, but it identifies subjects that suffer from mood disorders independently from the distress associated with AD [46]. Finally, it is widely reported in the literature how a greater severity of initial depressive symptoms is associated with their greater persistence in the long term, as outlined by the results of Buoli and co-authors’ study [47].

With regard to anxiety symptoms, the only factor that was found to be associated with the long-term persistence of these conditions is female gender. The observation of the higher vulnerability of those of the female gender to anxiety disorders is widely reported in literature, and it is supported by different findings, including (1) a higher prevalence of these disorders in women than men [48]; (2) the fact that women experience large fluctuations in the expression of estrogens, which are protective against psychiatric conditions [49,50]; and (3) the observation that women are more vulnerable to the detrimental effects of stress, such as those caused by a chronic illness like AD [51].

In conclusion, dupilumab ameliorates affective symptoms in patients affected by AD, but dermatologists should take special care of patients that can experience the negative effects of persistent psychiatric symptoms, such as women with overweight/obesity or subjects with severe depressive symptoms at the initiation of therapy. These findings imply that for specifics group of patients with AD, strict collaboration between the dermatologist and mental health professionals is needed to optimize treatment [52]. On the other hand, recovery from anxiety and mood disorders in patients suffering from AD is desirable for improving compliance with medical therapy, for promoting healthy lifestyles that include physical activity and a regular diet, and for improving social functioning, which, in turn, is associated with a reduction in stress which could exacerbate the dermatological disease [53].

This study has several limitations which should be noted: First of all, there was no randomization in the assignment of therapy with dupilumab, and there was no control group. Moreover, medical and psychiatric comorbidities [39] may have influenced the mental health of the sample, while previous pharmacological treatment may have contributed to the onset of affective disorders (e.g., systemic corticosteroids and mood symptoms). Finally, the context of the pandemic may have impacted on the mental health of fragile subjects, such as patients with AD [54,55]. With regard to this latter point, it is necessary to take into account that the Lombardy region was hardly hit by the pandemic in 2020–2021 and that a large part of the population was found to be positive via molecular swab at least once, considering all the COVID waves [56].

Randomized controlled studies with larger multi-centric samples are needed to confirm the preliminary results described in the present article. Specifically, to corroborate the effectiveness of dupilumab on affective disorders, the ideal study should consist of selecting samples of patients suffering from mood or anxiety conditions and without dermatological comorbidity to be randomized to treatment with the immune modulator versus first-line treatments such as Selective Serotonin Reuptake Inhibitors [24]. Since it is known that affective disorders can influence different biological systems, it would be interesting to measure a series of biochemical parameters in different treatment groups. The existing literature has, in fact, underlined how the different biological systems are in direct connection with each other and how they can influence the onset and course of mental disorders: for example, a state of over-inflammation is responsible for neurotransmitter alterations such as an accelerated metabolism of serotonin at the level of the Central Nervous System [57].

## Figures and Tables

**Table 1:** Baseline clinical variables of the total sample.

Variables	Total Sample (N = 331)
Age	37.03 (±16.96)
Age at onset	11.12 (±18.97)
Gender	
Male	183 (55.3%)
Female	148 (44.7%)
BMI	24.46 (±10.50)
Duration of illness	26.03 (±15.70)
Type of AD	
Extrinsic	300 (90.6%)
Intrinsic	31 (9.4%)
Baseline Systemic Treatment	
Cyclosporine	58 (17.5%)
Corticosteroids	26 (7.9%)
Methotrexate	5 (1.5%)
Antihistamines	7 (2.1%)
No systemic treatment	235 (71.0%)
Previous treatment with cyclosporine	
Yes	278 (84.0%)
No	53 (16.0%)
Previous treatment with corticosteroids	
Yes	258 (77.9%)
No	73 (27.1%)
Previous treatment with methotrexate	
Yes	30 (9.1%)
No	301 (90.9%)
Previous treatment with azathioprine	
Yes	7 (2.1%)
No	324 (97.9%)
Previous treatment with alitretinoin	
Yes	9 (2.7%)
No	322 (97.3%)

Legend: AD: Atopic Dermatitis; BMI: body mass index. Means for continuous variables and frequencies for qualitative ones are reported in the table. Standard deviations for continuous variables and percentages for qualitative ones are reported in brackets.

**Table 2:** Rating scale scores and IgE of the total sample at baseline and after 24 months of dupilumab treatment.

Variables	Baseline (N = 331) Mean (±SD)	24 Months (N = 331) Mean (±SD)	t	*p*
Total IgE (kU/L)	3619.48 (±6348.51)	655.95 (±1147.56)	6.23	<0.001
HADS-anxiety	8.46 (±4.23)	3.41 (±3.57)	18.14	<0.001
HADS-depression	7.22 (±4.23)	3.30 (±3.80)	14.35	<0.001
POEM	21.27 (±5.73)	5.56 (±5.14)	40.83	<0.001
DLQI	15.70 (±6.63)	2.61 (±2.98)	34.27	<0.001
SQ-NRS	6.81 (±3.07)	0.65 (±1.65)	33.64	<0.001
itch-NRS	8.49 (±1.42)	2.47 (±2.35)	40.06	<0.001
PGA	3.51 (±0.55)	0.77 (±0.74)	52.16	<0.001
EASI	29.07 (±7.21)	1.88 (±2.56)	65.27	<0.001
ADCT	19.98 (±3.81)	3.86 (±3.68)	54.82	<0.001

Legend: ADCT: Atopic Dermatitis Control Tool; DLQI: Dermatology Life Quality Index; EASI: Eczema Area and Severity Index; HADS: Hospital Anxiety and Depression Scale; IgE: immunoglobulin E; Itch-NRS: Itch Numerical Rating Scale; *p*: *p*-value.

**Table 3:** Binary logistic regression with presence of depressive symptoms at 24 months as dependent variable (HADS-D ≥ 8).

Baseline Variables	B	SE	Wald	*p*-Value	Exp(B)	95% CI for Exp(B)
Age	−0.002	0.017	0.019	0.889	0.998	0.966–1.031
Age at onset	−0.005	0.016	0.107	0.744	0.995	0.963–1.027
BMI	0.146	0.058	6.373	**0.012**	1.157	1.033–1.296
IgE	0.001	0.001	0.112	0.738	1.000	1.000–1.000
Gender	0.775	0.459	2.853	0.091	2.171	0.883–5.337
EASI	−0.042	0.038	1.261	0.261	0.959	0.891–1.032
PGA	−0.134	0.407	0.108	0.743	0.875	0.394–1.944
Itch-NRS	−0.214	0.167	1.653	0.199	0.807	0.582–1.119
SQ-NRS	0.150	0.098	2.344	0.126	1.162	0.959–1.407
DLQI	−0.108	0.044	5.933	**0.015**	0.898	0.823–0.979
POEM	0.038	0.049	0.619	0.431	1.039	0.944–1.143
ADCT	0.036	0.075	0.232	0.630	1.037	0.895–1.201
HADS-Depression	0.226	0.073	9.571	**0.002**	1.253	1.086–1.446
HADS-Anxiety	0.019	0.074	0.064	0.800	1.019	0.882–1.177
Extrinsic/intrinsic subtype	−0.347	0.734	0.223	0.637	0.707	0.168–2.981

ADCT: Atopic Dermatitis Control Tool; B: regression coefficient; BMI: body mass index; CI: confidence interval; DLQI: Dermatology Life Quality Index; EASI: Eczema Area and Severity Index; Exp: exponential; HADS: Hospital Anxiety and Depression Scale; IgE: immunoglobulin E; Itch-NRS: Itch Numerical Rating Scale; PGA: Physician Global Assessment; POEM: Patient-Oriented Eczema Measure; SE: standard error of B; SQ-NRS: Sleep Quality Numeric Rating Scale; Wald: Wald statistics. In bold are statistically significant values—*p* (≤0.05).

**Table 4:** Binary logistic regression with presence of anxiety symptoms at 24 months as dependent variable (HADS-D ≥ 8).

Baseline Variables	B	SE	Wald	*p*-Value	Exp(B)	95% CI for Exp(B)
Age	0.007	0.017	0.190	0.663	1.007	0.975–1.041
Age at onset	0.002	0.017	0.014	0.906	1.002	0.970–1.035
BMI	0.097	0.056	2.942	0.086	1.102	0.986–1.230
IgE	0.001	0.001	0.473	0.492	1.000	1.000–1.000
Gender	1.070	0.505	4.487	**0.034**	2.916	1.083–7.848
EASI	−0.049	0.039	1.565	0.211	0.953	0.883–1.028
PGA	0.276	0.446	0.383	0.536	1.318	0.550–3.160
Itch-NRS	−0.081	0.167	0.233	0.629	0.923	0.665–1.279
SQ-NRS	0.141	0.099	2.013	0.156	1.152	0.948–1.399
DLQI	−0.010	0.043	0.054	0.816	0.990	0.910–1.077
POEM	0.002	0.046	0.002	0.969	1.002	0.915–1.097
ADCT	−0.075	0.076	0.960	0.327	0.928	0.799–1.077
HADS-Depression	0.120	0.074	2.660	0.103	1.128	0.976–1.304
HADS-Anxiety	−0.076	0.078	0.951	0.329	0.927	0.795–1.080
Extrinsic/intrinsic subtype	−0.128	0.713	0.032	0.858	0.880	0.217–3.562

ADCT: Atopic Dermatitis Control Tool; B: regression coefficient; BMI: body mass index; CI: confidence interval; DLQI: Dermatology Life Quality Index; EASI: Eczema Area and Severity Index; Exp: exponential; HADS: Hospital Anxiety and Depression Scale; IgE: immunoglobulin E; Itch-NRS: Itch Numerical Rating Scale; PGA: Physician Global Assessment; POEM: Patient-Oriented Eczema Measure; SE: standard error of B; SQ-NRS: Sleep Quality Numeric Rating Scale; Wald: Wald statistics. In bold are statistically significant values—*p* (≤0.05).

## Data Availability

The data presented in this study are available upon request to the corresponding author.
